# Dysosmia and dysgeusia due to the 2019 Novel Coronavirus; a hypothesis that needs further investigation

**DOI:** 10.1186/s40902-020-00254-7

**Published:** 2020-03-30

**Authors:** Seied Omid Keyhan, Hamid Reza Fallahi, Behzad Cheshmi

**Affiliations:** 1 National Advance Center for Craniomaxillofacial Reconstruction, Tehran, Iran; 2 Craniomaxillofacial Research Center, Tehran University of Medical Sciences, Tehran, Iran; 3 Regenerative Medicine and Stem Cell Research Network, Shahid Beheshti University of Medical Sciences and Health Services, Tehran, Iran; 4 School of Advanced Technologies in Medicine, Shahid Beheshti University of Medical Sciences, Tehran, Iran; 5 Dental Research Center, Research Institute of Dental Sciences, Shahid Beheshti University of Medical Sciences, Tehran, Iran; 6 Faculty of Dentistry, Boroujerd Islamic Azad University, P.O. 6915136111, Boroujerd, Iran

Coronaviruses are known as enveloped viruses with a positive-sense single-stranded RNA genome that their helical symmetry nucleocapsid is about 26–32 kilobases in size, making it the largest investigated genome among RNA viruses. The disease caused by 2019 new coronavirus (2019-nCoV) was named coronavirus disease-19 (COVID-19) by the World Health Organization in February 2020. The 2019-nCoV is phylogenetically related to severe acute respiratory syndrome-coronavirus (SARS-CoV) [1]. It has been shown that 2019-nCov enters the cell through the ACE2 cell receptor in the same way as the severe acute respiratory syndrome (SARS) coronavirus [2]. 2019-nCoV effectively uses angiotensin-converting enzyme 2 receptor (ACE2) as a receptor for cell invasion [3]. Primary non-specific reported symptoms of 2019-nCoV infection at the prodromal phase are malaise, fever, and dry cough. The most commonly reported signs and symptoms are fever (98%), cough (76%), dyspnea (55%), and myalgia or fatigue (44%) [4].

Our information on the probability and effect of 2019-nCoV on the peripheral and central nervous system is still scarce and therefore unreliable. There have been various studies evaluating coronavirus’s effects on the central nervous system. These studies suggest that the human central nervous system (CNS) may be susceptible to coronavirus infection [5]. Routes intended for central nervous system infection with coronaviruses are peripheral trigeminal or olfactory nerves following intranasal inoculation [6, 7]. The findings of studies on rodents show that these viruses cause demyelination and stimulate T cell-mediated autoimmune reactions against CNS antigens producing the question about the relation between coronaviruses especially the 2019-nCoV and neurologic disorder in humans. Given that the peripheral trigeminal or olfactory nerves are pathways of penetration of the coronaviruses into the central nervous system, and based on animal studies, it may be hypothesized that complications such as demyelination and stimulation of T cell-mediated autoimmune reactions may occur in the path of the infection spreading, so the occurrence of dysosmia and dysgeusia can be considered potential consequences of these nerve injuries.

Numerous reports of loss of sense of smell and taste have been received from Iranian people as one of the most heavily involved countries with COVID-19 during the outbreak of the disease [8, 9]. Significant numbers of people with confirmed COVID-19 also reported a complete or partial loss of their sense of smell and taste in the early stages. Initial investigations also indicate that in some cases, if one member of a family has experienced such symptoms, other family members have experienced similar symptoms over a short period of time. Another primary point that needs further investigation is that in confirmed COVID-19 patients with reported dysosmia and dysgeusia, often, other manifestations were less severe and the patients frequently recovered more quickly. In addition to what has been said that require further investigations to establish their validity, the timeliness or permanence of these complications, as well as how they are likely to be managed and treated, are of particular importance and require thoughtful scrutiny.

Although there is not sufficient evidence to make a definitive judgment and need more comprehensive investigations, two scenarios are more likely to be suggested as the cause of such an incident. The appearance of dysosmia and dysgeusia whether can be attributed to olfactory nerve and trigeminal nerve damage caused by the 2019-nCoV infection or excessive exposure to chemicals and disinfectants that are more commonly used by people due to the viral epidemic. Designing a study to assess the validity of such a hypothesis is important in that it can be considered a relatively acceptable diagnostic criterion for both the individual and the physicians. Since the existence of such a relationship is likely, it also seems likely that during the COVID-2019 outbreak, those who experience complications such as dysosmia and dysgeusia should be considered potential carriers of the virus and that in addition to necessary hygiene measurements, they must quarantine themselves.
